# Human epidermal growth factor receptor 4 (HER4) is a favorable prognostic marker of breast cancer: a systematic review and meta-analysis

**DOI:** 10.18632/oncotarget.12485

**Published:** 2016-10-05

**Authors:** Jue Wang, Jun Yin, Qing Yang, Feng Ding, Xiao Chen, Bingjie Li, Xingsong Tian

**Affiliations:** ^1^ Department of Breast and Thyroid Surgery, Shandong Provincial Hospital Affiliated to Shandong University, Jinan, Shandong, China; ^2^ Department of Systems Biology, MD Anderson Cancer Center, Houston, Texas, USA; ^3^ Department of Breast and Thyroid Surgery, Shandong Provincial Hospital Affiliated to Shandong University, Jinan, Shandong, China, Department of General Surgery, Jinan Hospital, Jinan, Shandong, China; ^4^ Division of Epidemiology, School of Public Health, The University of Texas Health Science Center at Houston, Houston, Texas, USA

**Keywords:** HER4/ErbB-4, breast cancer, meta-analysis, prognosis, marker

## Abstract

Based on a large cohort of clinical studies involving a total of 8024 patients and reporting the effects of HER4 on breast cancer prognosis, we conducted the first meta-analysis and review of this type. We identified 26 studies published between 1985 and 2016 and assessed the prognostic value of HER4 in breast cancer by either real-time quantitative reverse transcription-polymerase chain reaction (RT-PCR, for mRNA levels) or immunohistochemistry (IHC, for protein levels). Elevated expression of HER4 was significantly associated with longer relapse-free survival (RFS) (HR = 0.63; CI: 0.48−0.83; *P* = 0.001, random effects). Further subgroup analysis showed that our results were stable irrespective of subtype [Luminal: HR = 0.40, CI: 0.30−0.53, *P* < 0.001, fixed effects; triple negative breast cancer (TNBC): HR = 0.49, CI: 0.26−0.90, *P* = 0.02, fixed effects; and HER2-positive: HR = 0.53, CI: 0.40−0.71, *P* < 0.001, fixed effects]. Cytoplasmic HER4 was more effective than nuclear HER4 (HR = 0.74, CI: 0.60−0.92, *P* = 0.007, fixed effects) for predicting RFS. HER4 was also found to be a favorable prognostic marker for overall survival (OS) among patients with non-TNBC in the subgroup analysis (Luminal: HR = 0.71, CI: 0.52−0.95, *P* = 0.023, fixed effects; HER2-positive: HR = 0.48, CI: 0.26−0.89, *P* = 0.020, fixed effects).

## INTRODUCTION

Breast cancer is one of the most frequently occurring malignancies and remains one of the leading causes of death in women. More than 1.7 million patients have been diagnosed with breast cancer since 2012 [1]. Current advanced therapies such as monotherapy, drug-targeted treatment (e.g., Herceptin) and endocrine treatment as well as traditional surgery, chemotherapy, and radiotherapy protocols are shifting breast cancer from incurable to chronically manageable.

The human epidermal growth factor receptor (EGFR) family, which includes EGFR (HER1 or ErbB1), HER2 (ErbB2), HER3 (ErbB3) and HER4 (ErbB4), is known for its crucial roles in carcinogenesis [2]. Therefore, members of this family have been applied as either biomarkers or drug targets in different types of cancers [2, 3]. Among all the EGFR family members, EGFR and HER2 have been well studied and found to be co-regulated in breast cancer. The emergence of the HER2-targeted antibody drug Herceptin/trastuzumab and the small dual molecule EGFR/HER2 kinase inhibitor Tykerb/lapatinib has remarkably improved the survival of patients with breast cancer; therefore, these drugs have been incorporated into the standard protocols for breast cancer treatment [3]. HER3 also plays a pivotal role in HER2-driven signaling by forming dimers with HER2 since HER3 lacks tyrosine kinase activity. Lipton et al. indicated that the protein expression level of HER3 could be used to define multiple subtypes of HER2-positive breast cancer, indicating the important but limited involvement of HER3 alone as a marker in breast cancer [4].

Compared with other members of the EGFR family, the function and prognostic capability of HER4 signaling are understudied and poorly understood. HER4 is known for its positive role in tumor progression, including the acceleration of human breast cancer cell growth [5, 6] and the induction of mouse mammary carcinoma formation [7]. HER4 can also transform *in vitro* and influence carcinomas in immune-deficient mice by interacting with other EGFR family members [8, 9]. Zhu et al. also indicated that HER4 alone could mediate estrogen-induced growth of breast cancer cells [10]. In contrast, activation or up-regulation of HER4 in breast cancer can significantly influence cell cycle arrest, differentiation and apoptosis *in vitro* [11, 12]. Overall, overexpression of HER4 is often detected in breast carcinomas, indicating the possible role of HER4 alone as either a diagnostic or prognostic marker for patients with breast cancer.

Although accumulating studies have attempted to associate HER4 expression with breast cancer prognosis, there is no consensus that HER4 is an advantageous prognostic marker of breast cancer since some of the existing studies have drawn controversial and opposing conclusions. To clarify the role of HER4 in the prognosis of breast cancer, we conducted this systematic review of the literature and performed a meta-analysis. We sought to determine whether high ErbB-4 mRNA levels or elevated/positive HER4 protein expression could be a prognostic marker for breast cancer.

## RESULTS

### Study selection and characteristics

**Figure 1:**
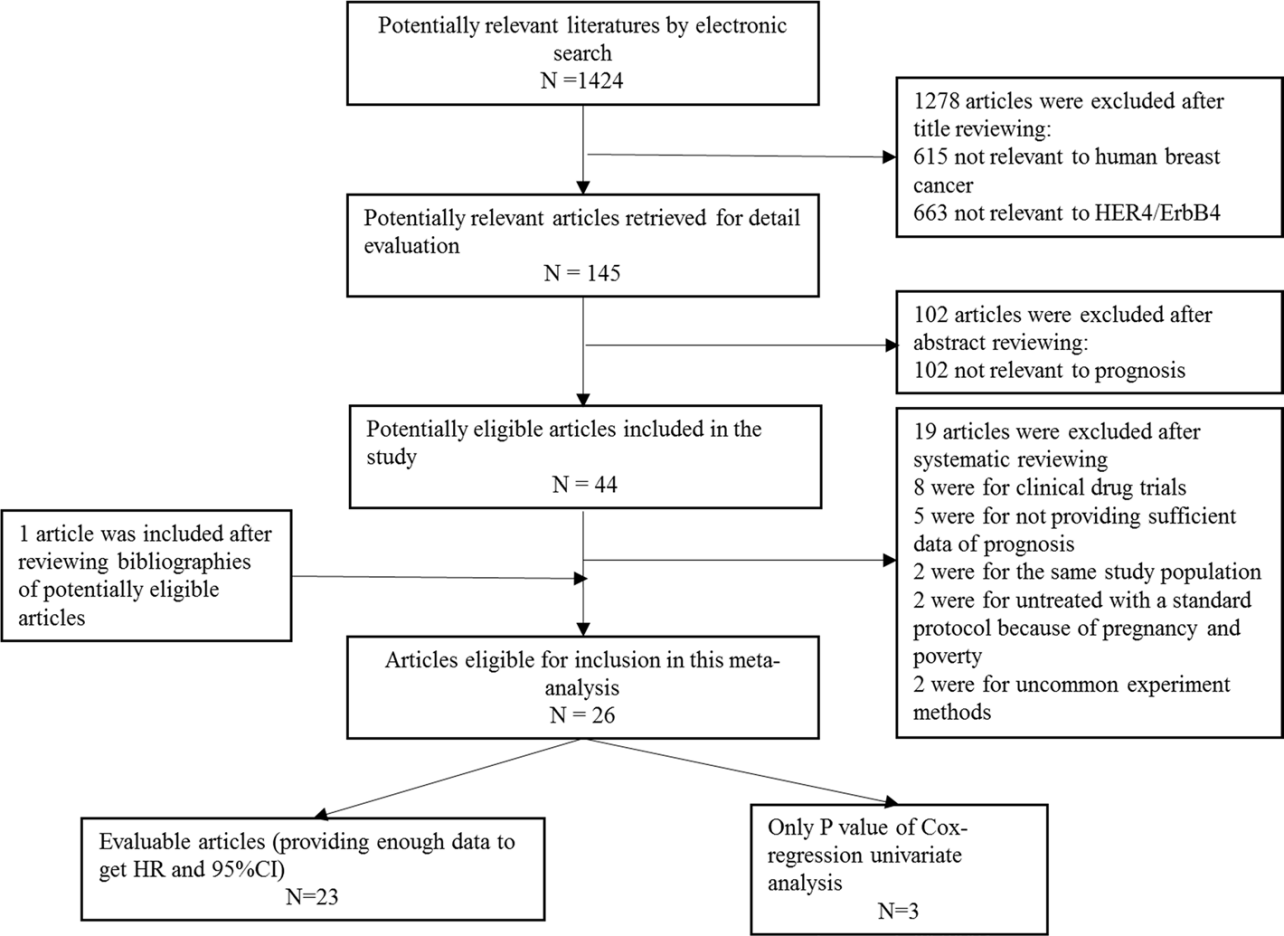
PRISMA flow chart of publication selection

After all duplicates were removed, 1424 studies were identified by a primary electronic literature search using MEDLINE, Embase and CNKI databases. However, due to either their irrelevance to human breast cancer, HER4/ErbB4, or breast cancer prognosis, 1380 studies were excluded. Forty-four studies were selected as the best candidates and were further reviewed in detail. After all 44 studies were further evaluated, 19 studies were removed for one of the following reasons: 1) the study included randomized clinical drug trials (*n* = 8); 2) the study had insufficient data reported for the prognostic analysis (*n* = 5); 3) the patients involved in the study did not receive a standardized treatment due to pregnancy and/or poverty (*n* = 2); 4) the study had duplicate publications based on the same patient cohorts (*n* = 2); or 5) the study involved other experimental methods other than immunohistochemistry (IHC) and real-time quantitative reverse transcription-polymerase chain reaction (RT-PCR) (*n* = 2). Additionally, we included one additional article from the reference lists of potentially eligible studies [13]. Finally, 26 studies [6, 13–37] were identified as eligible and were further analyzed (Figure 1).

The included 26 studies encompassed 8024 patients with breast cancer from 10 different countries and were published between 2000 and 2015. The majority of the studies (22/26) used IHC to identify the protein expression of HER4. The mRNA levels of HER4 were identified by RT-PCR in the rest of the qualifying studies (4/26). The main characteristics of the selected studies are shown in Table 1 (overall analysis) and Table 2 (sub-group analysis).

**Table 1:** Characteristics of studies used in overall analysis

First author	Journal	Published year	Country	Age (Median)	Follow-up Duration	Population Size	Stage	Lab Methods	Survival Indicators	Treatment
Valérie Pawlowski	Clinical Cancer Research	2000	France	26–90 (58)	Median: 77.6 months	365	N/A	rt-PCR	RFS, OS	Surgery, Adjuvant Chemotherapy, endocrine therapy, Radiotherapy: as protocols
Zhenhe Suo	Journal of Pathology	2002	N/A	32–90 (64)	11 years	100		IHC (Santa Cruz sc-283)	DFS, CSS	Surgery, Adjuvant Chemotherapy, endocrine therapy, Radiotherapy: as protocols
Caroline J Witton	Journal of Pathology	2003	UK	N/A	N/A	220	N/A	IHC (H4.77.16, Neomarkers)	BCSS	Surgery, Adjuvant Chemotherapy, endocrine therapy, Radiotherapy: standard treatment
Laboratoire d'Oncogénétique	International Journal of Cancer	2003	France	31–91 (58.2)	Median: 8.1 years	130	N/A	rt-PCR	RFS	N/A
DM Abd El-Rehim	British Journal of Cancer	2004	UK	18–70 (53)	Median: 58 months	1944		IHC (HFR-1 antibody,NeoMarkers)	DFS, OS	N/A
Nicola L.P. Barnes	Clinical Cancer Research	2005	UK	39–82 (55.5)	5 years	129	DCIS	IHC (Santa Cruz sc-283)	DFS	Surgery & Adjuvant radiotherapy
Teemu T. Junttila	Cancer Research	2005	Finland	N/A	Median: 10 years	458		IHC (HFR-1 antibody,NeoMarkers)	DFS	Surgery & Adjuvant radiotherapy, Adjuvant endocrine therapy
Sam M. Wiseman	Cancer	2005	Canada	N/A	Median: 15 years	242	I–III	IHC (HFR-1 antibody,NeoMarkers)	DSS	N/A
ILKA B. FUCHS	Anticancer Research	2006	Germany	N/A	240 months	48		IHC (C-18,Santa Cruz)	OS	Surgery, Neo/Adjuvant Chemotherapy, endocrine therapy: as protocols
M Aubele	British Journal of Cancer	2007	Germany	27–84 (66)	Median: 144 months	193	N/A	IHC (H4.77.16, Neomarkers)	EFS	Surgery, Adjuvant Chemotherapy, endocrine therapy, Radiotherapy: as protocols
Andrea Sassen	Breast Cancer Research	2008	Germany	25–82 (55)	Median: 125.6 months	278		IHC (Cell Signaling 83B10)	DFS, OS	N/A
M Aubele	British Journal of Cancer	2008	Germany	N/A	Median: 80 months	426	N/A	IHC (H4.77.16, Neomarkers)	DFS	Surgery, Adjuvant Chemotherapy, endocrine therapy, Radiotherapy: as protocols
Anjali Naresh	Cancer Research	2008	US	N/A	Median: 15.6 years	42	N/A	IHC (HFR-1 antibody,NeoMarkers)	DSS	Surgery, Chemotherapy, Radiotherapy, Endocrine therapy
Thomas Frogne	Breast Cancer Research	2009	Denmark	48–74 (61)	N/A	268		IHC (Thermo Fisher Scientific RB-9045)	DFS, OS	Surgery & Adjuvant endocrine therapy
Ann D. Thor	The American Journal of Pathology	2009	US	N/A	Median: 15.6 years	923	N/A	IHC (HFR-1 antibody,NeoMarkers)	DFS, DSS	Surgery, Chemotherapy, Radiotherapy
Emmet McIntyre	Breast Cancer Res Treat	2009	UK	27–73	195 months	100	N/A	IHC (HFR-1 antibody)	OS	Standard protocols
Ling Zhang	ACTA UNIVERSITATIS MEDICINALIS NANJING (Natural Science)	2011	China	22–70 (43)	N/A	105	I–III	IHC	DFS	Surgery & Adjuvant chemotherapy
Tanja Badovinac-Crnjevic	Medical Oncology	2011	Croatia	N/A	Median: 60 months	181	N/A	IHC (Abcam,clone SPM338)	DFS, OS	N/A
B M Syed	British Journal of Cancer	2013	UK	> 75	36 years	575		IHC (H4.77.16, Neomarkers)	DFS, BCSS	Surgery & Adjuvant radiotherapy, Adjuvant endocrine therapy
Junichi Kurebayashi	Breast Cancer	2013	Japan	24–83 (54)	Median: 38.5 months	87	N/A	IHC (Thermo Fisher Scientific)	RFS	Surgery, Target therapy, Adjuvant chemotherapy
Anna Machleidt	BMC Cancer	2013	Germany	24–83 (54)	N/A	172		rt-PCR	EFS, OS	Surgery, Target therapy, Adjuvant chemotherapy, Endocrine therapy
K. Hashimoto	Annals of Oncology	2014	Japan	28–82 (56)	N/A	75		IHC	EFS, OS	Surgery & Adjuvant chemotherapy
Saori Fujiwara	Breast Cancer	2014	Japan	21–93 (59)	120 months	250	N/A	rt-PCR	DFS, BCSS	Surgery, Neo/Adjuvant Chemotherapy, endocrine therapy: as protocols
Siti Norasikin Mohd Nafi	Oncotarget	2014	UK	N/A	N/A	73	N/A	IHC[antibodies against c-terminus HER4 (Santa Cruz), c-terminus HER4 (Neomarkers)]	OS, RFS	Neoadjuvant chemotherapy and trastuzumab treatment

N/A: not available.

**Table 2:** Characteristics of studies involved in sub-group analysis

First author	Journal	Published year	Country	Follow-up Duration	Molecular Type	Stage	Lab Methods*	Survival Indicators	Treatment
Zhenhe Suo	Journal of Pathology	2002	N/A	11 years	Her2- positive	I-IV	IHC	DFS, CSS	Surgery, Adjuvant Chemotherapy, endocrine therapy, Radiotherapy: as protocols
Caroline J Witton	Journal of Pathology	2003	UK	N/A	N/A	N/A	IHC	BCSS	Surgery, Adjuvant Chemotherapy, endocrine therapy, Radiotherapy: standard treatment
Nicola L.P. Barnes	Clinical Cancer Research	2005	UK	5 years	Her2- positive	DCIS	IHC	DFS	Surgery & Adjuvant radiotherapy
Teemu T. Junttila	Cancer Research	2005	Finland	Median: 10 years	Luminal	I	IHC	DFS	Surgery & Adjuvant radiotherapy, Adjuvant endocrine therapy
Sian M Tovey	Breast Cancer Research	2006	UK	Median: 6.45 years	Luminal	N/A	IHC (Nuclear,Cytoplasm)	BCSS	Surgery, Adjuvant endocrine therapy: standard treatment
Anjali Naresh	Cancer Research	2008	US	Median: 15.6 years	Luminal	N/A	IHC	DSS	Surgery, Chemotherapy, Radiotherapy, Endocrine therapy
Thomas Frogne	Breast Cancer Research	2009	Denmark	N/A	Luminal	I-IV	IHC	DFS, OS	Surgery & Adjuvant endocrine therapy
Ann D. Thor	The American Journal of Pathology	2009	US	Median: 15.6 years	N/A	N/A	IHC (Nuclear,Cytoplasm)	DFS, DSS	Surgery, Chemotherapy, Radiotherapy
Ling Zhang	ACTA UNIVERSITATIS MEDICINALIS NANJING (Natural Science)	2011	China	N/A	N/A	I–III	IHC	DFS	Surgery & Adjuvant chemotherapy
Junichi Kurebayashi	Breast Cancer	2013	Japan	Median: 38.5 months	Her2- positive	N/A	IHC	RFS	Surgery, Target therapy, Adjuvant chemotherapy
Anna Machleidt	BMC Cancer	2013	Germany	N/A	TNBC, HER2-positive, Luminal A	I-IV	rt-PCR	EFS, OS	Surgery, Target therapy, Adjuvant chemotherapy, Endocrine therapy
K. Hashimoto	Annals of Oncology	2014	Japan	N/A	TNBC	I-IV	IHC (Nuclear, Membrane, Cytoplasm)	EFS, OS	Surgery & Adjuvant chemotherapy
Siti Norasikin Mohd Nafi	Oncotarget	2014	UK	N/A	Her2- positive	N/A	IHC (Nuclear,Cytoplasm)	OS RFS	Neoadjuvant chemotherapy and trastuzumab treatment
Saori Fujiwara	Oncotarget	2014	Japan	Median: 65 months	Luminal A	N/A	IHC (Nuclear,Cytoplasm)	DFS	Standard protocols

* if the article was belong to the sub-group of different localization of HER4 expression, the localization would be noted.

### Quality assessment

The main characteristics of all potentially eligible studies was shown in Supplementary Table S1 in File S1. Based on the European Lung Cancer Working Party (ELCWP) scoring scale [38], the overall quality assessment of the selected studies ranged from 63.75% to 85% with a median of 76.25% (Suppelemntary Table S2A in File S1, mean and SD values are shown). No significant discrepancies were detected between the 26 qualifying studies and the excluded studies (*P* = 0.079), and no significant difference was observed between the scores of the positive studies and negative studies (*P* = 0.091). Additionally, there existed no significant difference between the significant and insignificant studies (*P* = 0.224) among the qualifying studies. The scores of the 26 studies included for meta-analysis are shown in Supplementary Table S2B in File S1 (Supplmentary Table S1 and Supplementary Table S2B).

### Global analysis of the influence of HER4/ErbB4 on prognosis

**Figure 2:**
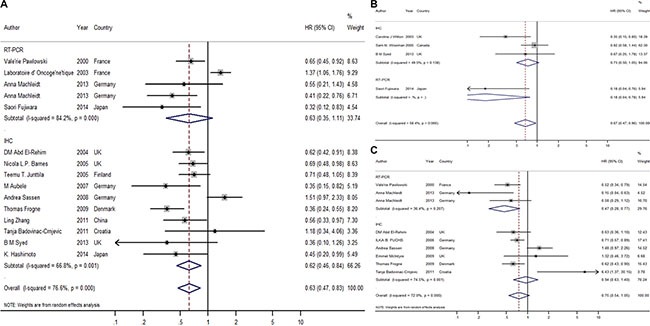
Forest plot of overall meta-analysis (**A**) Overall meta-analysis of RFS; (**B**) Overall meta-analysis of BCSS; (**C**) Overall meta-analysis of OS.

A total of 17 qualifying studies used disease-free survival (DFS), relapse-free survival (RFS) or event-free survival (EFS) as the endpoint. Hazard ratios (HRs) of DFS/RFS/EFS from 14 articles involving 5144 patients [6, 14–17, 19–21, 23, 25, 27–32, 37] were either extracted or calculated for overall analysis. The majority of the studies (*n* = 10) used IHC to evaluate the protein level of HER4; these studies included 4227 patients. The other four studies involved 917 samples and used RT-PCR to detect the mRNA levels of ErbB4. The estimated pooled HRs for these 14 studies revealed that elevated/positive HER4 expression played a more favorable role in the RFS of patients with breast cancer (HR = 0.63; CI: 0.48–0.83; *P* = 0.001, random effects; Figure 2A). The test of heterogeneity was significant (*P* < 0.001, *I*^2^ = 75.6%). We further evaluated the prognostic effects of protein levels (as measured by IHC) and mRNA levels (as measured by RT-PCR) of HER4 separately. The protein levels of HER4 were positively associated with RFS (HR = 0.62; CI: 0.46–0.84, random effects), but the mRNA levels of HER4 were not (HR = 0.63; CI: 0.35–1.11, random effects). This finding indicated that the protein level, rather than the mRNA level, of HER4 could be used as an effective marker for RFS. When 3 other studies [14, 35, 36] with firm conclusions but no evaluable HRs were included, approximately 71% of the selected studies indicated that HER4 expression correlated with a better RFS.

The estimated HRs for breast cancer-specific survival (BCSS)/disease-specific survival (DSS)/cancer-specific survival (CSS) were available in 6 publications, which included 1429 samples [16–18, 21, 24, 35]. Further estimated pooled HRs showed that elevated/positive HER4 expression was a favorable marker for BCSS with no heterogeneity (HR = 0.67, CI: 0.47–0.96, *P* = 0.028, fixed effects; *P* = 0.066, *I*^2^ = 58.4%; Figure 2B). Half of all of the selected studies indicated a favorable association between HER4 and BCSS.

The estimated HRs for overall survival (OS) were available in 8 studies [19, 20, 22, 23, 30, 32, 34, 37] involving 3356 patients. No significant association was observed between the high/positive expression of HER4 and OS (HR = 0.75; CI: 0.54–1.05; *P* = 0.10, random effects; Figure 2C). Considering that significant heterogeneity was found (*P* < 0.001, *I*^2^ = 72.1%), the random effects model was applied. Based on the meta-analysis of the different methods, the result indicated that mRNA levels of HER4 could be a favorable marker for OS (HR = 0.47; CI: 0.28–0.77) with homogeneity. This result indicated that different expression levels of HER4 (either protein or mRNA; in this case, mRNA) could be selectively used for breast cancer prognosis depending on the endpoint selected for the study.

### Influences of HER4 on the survival of different molecular subtypes of breast cancer

In general, breast cancer includes 3 major molecular subtypes (Luminal, TNBC, and HER2+), which can be separated by their gene expression profiles. Mounting evidence has demonstrated that divergent molecular subtypes have remarkable differences in terms of their histology, molecular alteration, therapeutic response and patient outcome. Therefore, the association of HER4 expression with different breast cancer molecular subtypes may provide more detailed and precise prognostic significance.

**Figure 3:**
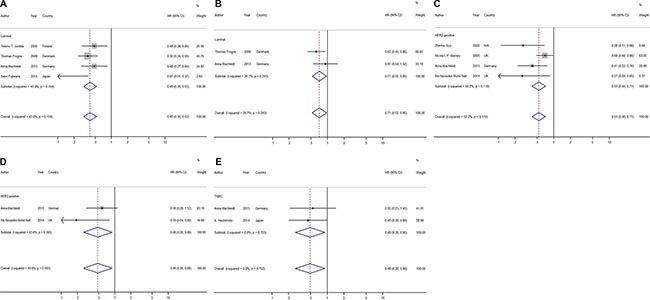
Forest plots of meta-analysis based on different molecular types of breast cancer (**A**) Meta-analysis on RFS of Luminal type; (**B**) Meta-analysis on OS of Luminal type; (**C**) Meta-analysis on RFS of HER2-positive type; (**D**) Meta-analysis on OS of HER2-positive type; (**E**) Meta-analysis on RFS of TN type.

In our analysis, the HRs for RFS were available in 4 studies reporting on Luminal breast cancer, which included 717 patients [6, 20, 36, 37]. The estimated pooled HR indicated that HER4 expression was associated with a significantly better prognosis for the Luminal subtype using RFS as the endpoint (HR = 0.40; CI: 0.30–0.53; *P* < 0.001; fixed effects; Figure 3A). No significant heterogeneity was detected for this subgroup *(P* = 0.154, *I*^2^ = 43.0%). Although there were only 2 studies providing evaluable HRs for OS, the pooled HR implied that patients with Luminal breast cancer would benefit from high expression of HER4 with no significant heterogeneity detected (HR = 0.71, CI: 0.52–0.95, *P* = 0.020, fixed effects; *P* = 0.243, *I*^2^ = 26.7%; Figure 3B).

The HER2-positive molecular subtype as we have defined here is different from the HER2+ subtype as we only considered expression of HER2 regardless of the ER/PR status. The HRs for RFS were available in 4 studies regarding the association of elevated/positive HER4 expression with HER2-positive breast cancer; these studies included 280 patients. Both IHC (3/4) and RT-PCR (1/4) were used in the evaluable studies [14, 21, 26, 37]. The estimated pooled HRs implied that the elevated/positive expression of HER4 was a favorable indicator of RFS in HER2-positive breast cancer (HR = 0.53, CI: 0.40–0.71, *P* < 0.001, fixed effects; Figure 3C), and there was no statistical significance in the heterogeneity test (*P* = 0.11, *I*^2^ = 50.0%). Two of the evaluable studies used OS as the endpoint. The estimated pooled HRs implied that HER4 was a favorable marker for OS with no significant heterogeneity (HR = 0.48, CI: 0.26–0.89, *P* = 0.020, fixed effects; *P* = 0.18, *I*^2^ = 43.6%; Figure 3D).

Two studies reported the effects of HER4 expression on TNBCs, which included 151 patients [15, 37]. Both of the studies used RFS and OS as endpoints, but only one article provided an evaluable HR for OS. A favorable prognosis was found on RFS in the group with high/positive expression of HER4 with no heterogeneity (HR = 0.49, CI: 0.26–0.90, *P* = 0.02, fixed effects; *P* = 0.75, *I*^2^ = 0%; Figure 3E). The only study that used OS suggested that high/positive HER4 expression tended to have an advantageous influence on overall survival (HR = 0.15; CI: 0.01–0.7; *P* = 0.01).

### Impact of the subcellular localization of HER4 on breast cancer survival

Unlike other members of the EGFR family, HER4 has its unique mechanism when undergoing the activation of its receptor. Activation of the HER4 receptor would lead to the release of the soluble HER4 intercellular domain (4ICD). This released portion might localize to either the cytosol to mitochondria to mediate tumor cell apoptosis [39, 40] or the nucleus to function as a possible co-activator of ER (estrogen receptor)-alpha and signal transducer and activator of transcription 5A [10, 41, 42], further regulating cell proliferation and differentiation. Therefore, an association of HER4 localization, if any, would contribute to the correlation of HER4 function with breast cancer survival.

**Figure 4:**
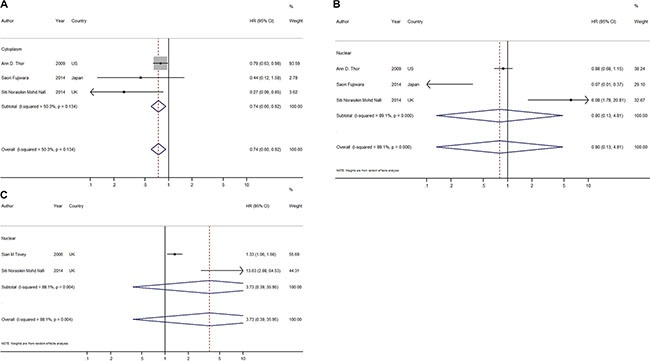
Forest plots of meta-analysis based on HER4 expression of different subcellular localization (**A**) Meta-analysis on RFS of HER4 expression in cytoplasm; (**B**) Meta-analysis on RFS of HER4 expression in nuclear; (**C**) Meta-analysis on OS of HER4 expression in nuclear.

In our review, 5 studies investigated HER4 nuclear expression; these studies included 1548 patients [13, 15, 26, 33, 36]. Four of the 5 studies (1207 patients) [15, 26, 33, 36] also provided information on HER4 cytoplasmic expression. However, we failed to detect a significant association between positive nuclear expression of HER4 and the prognosis of breast cancer with regard to both OS (OS: HR = 3.73, CI: 0.39–35.95, *P* = 0.25, random effects; *P* = 0.004, *I*^2^ = 88.1%; Figure 4C) and RFS (RFS: HR = 0.80, CI: 0.13–4.81, *P* = 0.80, random effects; *P* < 0.001, *I*^2^ = 89.1%; Figure 4B). In contrast, HER4 expression in the cytoplasm was indicated to have an advantageous effect on RFS with no significant heterogeneity (HR = 0.74, CI: 0.60–0.92, *P* = 0.007, fixed effects; *P* = 0.13, *I*^2^ = 50.3%; Figure 4A). Among all the studies included in this meta-analysis, we were able to draw a conclusion from 75% of them, which indicated that elevated cytoplasmic expression of HER4 was related to a prolonged RFS. The one available study reporting the HRs for OS and BCSS indicated that cytoplasmic expression also played a positive role in OS (HR = 0.19, CI: 0.04–0.8, *P* = 0.0024) but not BCSS (HR = 0.79, CI: 0.61–1.02, *P* = 0.07).

### Publication bias

**Figure 5:**
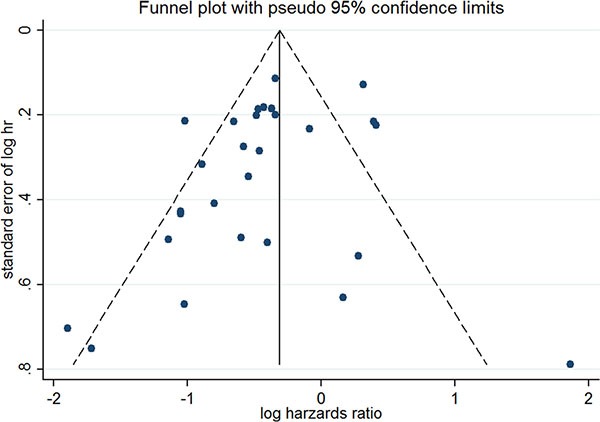
The funnel plot with pseudo 95% confidence interval of all the evaluable publications

After performing Begg's linear regression model, no publication bias was found among all of the available studies in the overall meta-analysis (Begg's test, *P* = 0.206) and among the three subgroups based on the different prognosis indicators (RFS: *P* = 0.216; BCSS: *P* = 0.174; OS: *P* = 0.835; Begg's test), indicating the stability of our results. A funnel plot with the pseudo 95% CIs of all of the evaluable publications is shown in Figure 5.

## DISCUSSION

This meta-analysis of the pooled data provided evidence that the high/positive expression of HER4 was significantly associated with a better prognosis in terms of RFS regardless of the molecular subtype of breast cancer and implied the importance of HER4 in predicting the OS of patients with non-TNBC. This meta-analysis further suggested that the presence of elevated HER4 expression in the cytoplasm was associated with good prognosis in terms of RFS with no heterogeneity.

Current therapies for breast cancer have remarkably improved patient survival, leading to prolonged follow-ups for survival endpoints, especially for the duration of OS. Changes in the physiological condition of patients after tumor progression would complicate the evaluation of OS and further weaken the impacts of a single factor (in this case, HER4) on OS. In our study, although OS, RFS and BCSS were all evaluated, RFS and BCSS would be more appropriate endpoints for evaluating the importance of HER4 on predicting patient prognosis than OS because both RFS and BCSS have more emphasis on breast cancer-related outcomes.

Considering the heterogeneity found in the overall analysis, the expression of different isoforms of HER4 might be one potential explanation. It is known that HER4 consists of three independent domains: a glycosylated extracellular ligand-binding domain, a single transmembrane domain and an intracellular domain (ICD) [39]. Four HER4 isoforms are generated by alternative splicing, which differ in either the juxtamembrane (JM) or cytoplasmic (CYT) domains. The difference between the JM-a and JM-b isoforms is that the former contains a proteolytic cleavage site for tumor necrosis factor-α converting enzyme (TACE) and consequently is able to be processed by γ-secretase at the transmembrane (TM) region. Fujiwara et al. had found that in breast cancer, the JM-a isoform was predominantly expressed rather than JM-b, which could not even be detected. Meanwhile, a higher JM-a/JM-b ratio indicated an association with a lower nuclear grade, ER and progesterone receptor (PR) positivity, HER2 negativity and a lower Ki-67 status, which to some extent demonstrated that JM-a played a pivotal role in carcinogenesis of breast tissue [36]. The difference between CYT-1 and CYT-2 is that the former includes a 16 amino acid sequence that contains both a PI3-K binding site (YTPM) and an interaction motif (PPXY) for the Itch E3 ubiquitin ligase. Based on this difference, these two isoforms differed in their ubiquitylation and kinase activity. Because of the YTPM site, CYT-1 could activate the PI3K-AKT pathway to escape apoptosis, induce proliferation and reduce differentiation [43, 44], which indicated that CYT-1 promoted breast carcinogenesis. Similar results were found in the study of Fujiwara et al., in which the CYT-2 dominant group had a significant correlation with better RFS than the CYT-1 dominant group [36]. According to the discussion above, the expression of different HER4 isoforms might be an important reason for the heterogeneity detected in the overall analysis.

Considering RFS in the overall analysis, different definitions of “high” mRNA level might be another reason for the significant heterogeneity. After reviewing and comparing the experimental method of every study, we noted that the cutoff for “high” levels of mRNA varied. The majority of studies used the median value as a cutoff within the tumor samples; however, some of the studies defined the “high” level by comparing the tumor samples with normal breast tissue.

Obviously, our study had other limitations. Although our meta-analysis was conducted according to the published results available worldwide from 1985–2016 with non-overlapping patients, it also prevented us from obtaining the individual patients' updated data. The updated data could have provided estimates that are more accurate and might have significantly reduced the error. Moreover, the small sample size in the subgroup analyses is a concern. As HER4 has been ignored in the past, we could not include more sufficient studies for our analysis, especially for the subgroup analyses. To some extent, limited studies would lead to a less convincing conclusion.

In conclusion, our study demonstrated and highlighted the strong prognostic value of HER4 in breast cancer carcinoma. The detection of the strong association of cytoplasmic HER4 with RFS might serve as an effective tool for investigating the multifunctionality of HER4 in predicting prognosis and its possible contribution to providing optimal treatment to patients with cancer. To achieve greater clinical value and utility of HER4 in breast cancer, more detailed identification of the expression and localization of HER4 isoforms should be initiated and validated in a broader cohort of patients with breast cancer, which would provide a much larger sample size and would be more informative.

## MATERIALS AND METHODS

### Search strategy

Three systematic literature searches were performed in Embase, MEDLINE and CNKI (China National Knowledge Infrastructure). Full-text studies published in either Chinese or English from January 1985 to March 2016 were used for this review and analysis. The following key words were included in the search: “breast cancer”; “breast carcinoma”; “breast neoplasm”; “breast malignancy” or “breast tumor”; “HER4” or “ErbB4”; and “surviv*”, “prognos*”, “marker”, “indicator” or “outcome”. To include more sufficient studies, we used less specific key words such as “EGFR family” and “T1GFR (type 1 growth factor receptor) family” to broaden our search. Among all the identified studies, only peer-reviewed journals were included, as letters and meeting abstracts were ineligible. Additionally, we reviewed the bibliographies of potential eligible reports and review articles of HER4 to supplement our study.

### Eligibility criteria

The available studies were counted toward our analysis if they reported prognosis data in patients with breast cancer stratified by their HER4/ErbB4 status (overexpression or positive expression of HER4 protein and high levels of HER4 mRNA) and if they provided adequate data for calculating an estimate of the hazard ratio (HR) and a 95% confidence interval (CI). Eligible studies that only provided P-values from a Cox regression univariate analysis were also included in our analysis. Articles including the prognostic data of patients who did not obtain a standardized treatment because of pregnancy or poverty status were not taken into consideration. Prognostic data based on randomized clinical drug trials were also ruled out since the studies of interest should be retrospective.

Among all the methods used to detect the expression level of HER4/ErbB4, the most commonly used experimental methods were incorporated, including IHC and RT-PCR. To avoid overlapping patient samples, only the most complete or the most recent studies were included in our analysis. The endpoints of prognosis were DFS/RFS/EFS, OS and BCSS/DSS/CSS.

### Data extraction and methodological assessment

Two authors (Jue Wang and Jun Yin) completed independent reviews of 1424 studies. According to the eligibility criteria, a total of 26 studies were selected and further reviewed. For each included study of sufficient quality, the following data were extracted: authors, publication year, publication journal, geographic location, follow-up duration, population size, breast cancer molecular type, stage, experimental methods (for IHC, the antibodies used), survival indicator and treatment. We also recorded the PFS/DFS/EFS, OS, or BCSS/DSS/CSS; survival curves; HR; and 95% CI if available. To avoid bias in the data collection process, three reviewers (Jue Wang, Jun Yin, and Qing Yang) extracted the data separately and subsequently compared the results. Selected studies were examined for internal consistency, and the discordance was resolved by discussion.

Three investigators (Jue Wang, Jun Yin, Bingjie Li) reviewed and scored each study independently according to the European Lung Cancer Working Party (ELCWP) scoring scale with some modifications as described in Method S1 in File S1 [38]. A consensus value for each study was reached by at least two investigators if there was a discrepancy in scoring from three investigators. The evaluation of the methodology included four main categories: design (scale of 1 to 10, 1 as the worst and 10 as the best), laboratory methods (scale of 1 to 10, 1 as the worst and 10 as the best), outcome generalizability (scale of 1 to 10, 1 as the worst and 10 as the best) and data analysis (scale of 1 to 10, 1 as the worst and 10 as the best). We used a percentage from 0 to 100% to show the final outcome, with a high score indicating good methodological quality.

### Statistical analysis

A meta-analysis was performed to compare the difference between HER4/ErbB4 expression status (high/positive expression vs. low/negative expression) using RFS, BCSS and OS as the endpoints. A *P*-value of 0.05 or less was considered statistically significant.

The significant difference between the distribution of the quality scores was evaluated using Mann-Whitney tests. The HR and 95% CI of each study were either collected from the original article directly if available or approximately calculated as suggested by Tierney et al. [45]. The pooled HR was obtained by either fixed- or random-effects models. When there was heterogeneity among the studies, random-effects estimates were used for further analysis. An observed/estimated HR < 1 indicated more favorable survival with high/positive HER4/ErbB4 expression. Studies with only *P*-values from Cox regression analyses available were categorized into ‘positive’ (favorable, *P* ≤ 0.05) or ‘negative’ (non-favorable, *P* > 0.05). These studies further contributed to an overall estimated percentage of ‘positive’ studies with a more sufficient sample size.

Heterogeneity among the studies was explored using Cochran's heterogeneity test, and the Knapp-Hartung Variance Estimator was used as the residual heterogeneity estimator for our analysis. We used a funnel plot and Begg's test to evaluate if there was any possible publication bias [46]. A *P*-value of 0.05 or less (two-sided *t*-test) was considered an indicator of significance for Begg's test.

All of the statistical analyses were conducted using STATA version 13.0 (Stata Corporation, College Station, TX). Studies eligible for a systematic review were defined as “eligible”, and studies providing sufficient data of an HR and 95% CI for our meta-analysis were defined as “evaluable”.

## SUPPLEMENTARY MATERIALS METHODS AND TABLE


